# Stimulation-Guided AAV Delivery and Longitudinal Assessment of Optogenetic Expression in Rat Motor Nerves

**DOI:** 10.21769/BioProtoc.5545

**Published:** 2025-12-20

**Authors:** Emma M. Moravec, Jordan J. Williams

**Affiliations:** 1 Joint Department of Biomedical Engineering, Marquette University and Medical College of Wisconsin, Milwaukee, WI, USA; 2 Department of Neurosurgery, Medical College of Wisconsin, Milwaukee, WI, USA

**Keywords:** Optogenetics, Adeno-associated virus, Intramuscular injection, Motor stimulation, Electromyogram analysis, Immunohistochemistry, Irradiance

## Abstract

Optogenetic stimulation of peripheral motor nerves is a promising technique for modulating neural activity via illumination of light-sensitive ion channels known as opsins. Stimulating muscle activity through this method offers many advantages, such as a physiological recruitment order of motor units, reduced fatigue, and target-specific stimulation, which make it a favorable option for use in many neuroscience and motor rehabilitation applications. To enable such optical stimulation, opsin expression in peripheral nerves can be achieved either with transgenic animal models or through injection of viral vectors. In this protocol, we describe a method for driving peripheral nerve opsin expression via intramuscular adeno-associated virus (AAV) injection with the goal of enhancing virus uptake by targeting injections to neuromuscular junctions with electrical stimulation. We also describe procedures for non-invasively assessing functional opsin expression over time with transdermal optical stimulation of opsin-labeled nerves and electromyography (EMG) recordings. The presence of time-locked EMG spikes 4–8 ms after each stimulation pulse demonstrates that functional opsin expression is present at a given assessment time point. Onset of functional optical sensitivity generally occurs 2–4 weeks following virus injection, and sensitivity generally peaks or plateaus between 6–10 weeks. Stimulation sequences such as light intensity, stimulation pulse width, and frequency sweeps provide further information on functional opsin expression at the testing timepoint. The methods presented here can be used for driving functional opsin expression with a standard AAV6 vector commonly used in similar experiments or as a protocol for assessing peripheral nerve opsin expression with novel viral vectors.

Key features

• Uses electrical stimulation to guide needle placement during intramuscular viral injection.

• Drives robust and muscle-specific opsin expression in peripheral motor neurons.

• Describes transdermal optical stimulation sequences with varying stimulation light intensity, pulse width, and frequency for longitudinal assessment of opsin expression.

• Adaptable for use with multiple viral vectors and target muscles.

## Graphical overview


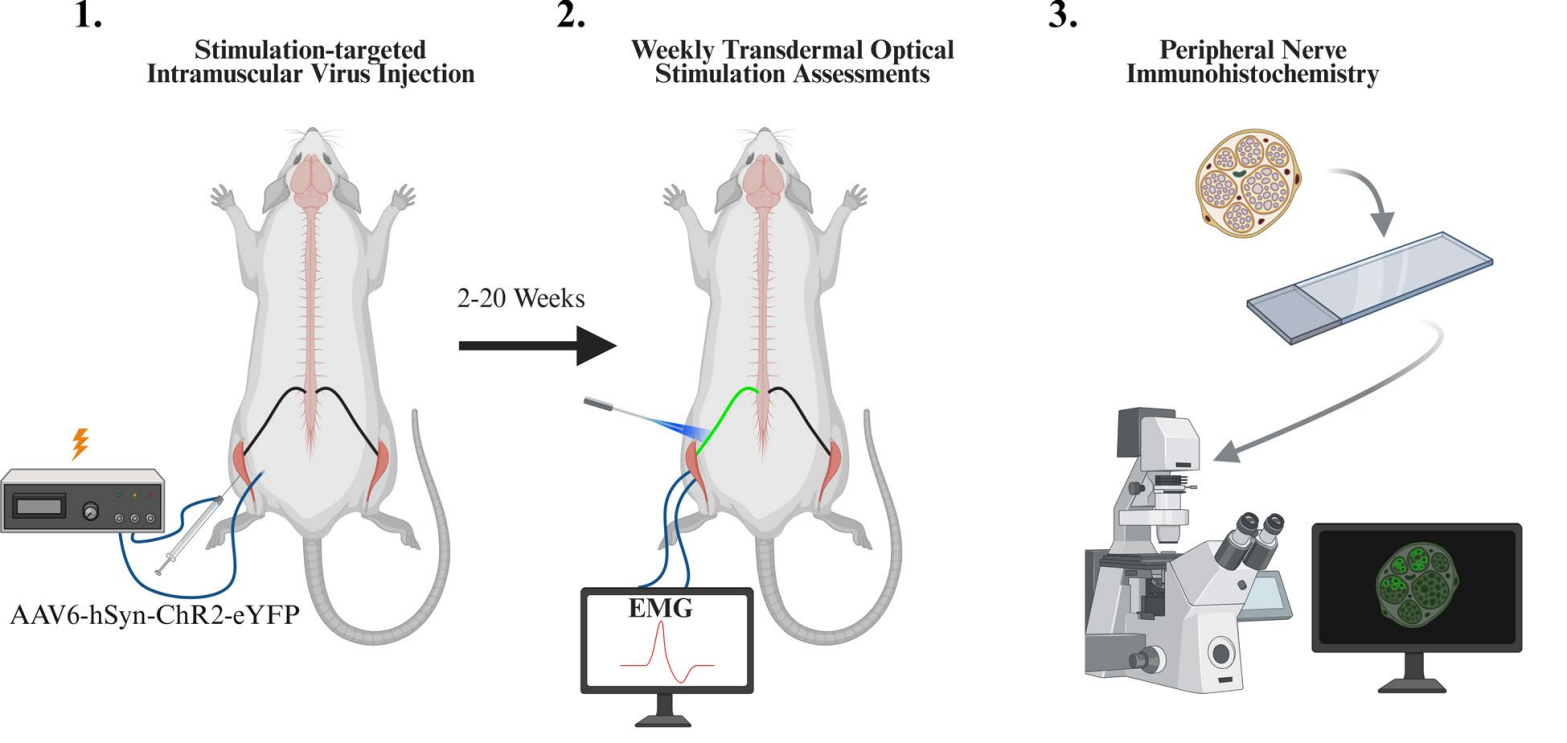


**Overview of experimental protocol including intramuscular viral injection, weekly optical stimulation sessions, and immunohistochemistry**

## Background

Optogenetic stimulation or inhibition of peripheral motor nerves has recently emerged as an approach to directly modulate muscle activity in animal models from rodents to non-human primates [1–4]. In this approach, targeted nerves are genetically modified with light-gated ion channels (“opsins”) such as the common channel-rhodopsin2 (ChR2), allowing a nerve to be stimulated or inhibited with specific light wavelengths. Compared to electrical stimulation, this approach has been found to confer benefits, including a more physiological recruitment order of motor units (i.e., small muscle fibers are first recruited with low-intensity optical stimulation, and larger muscle fibers are added at higher intensities) and reduced stimulation-induced muscle fatigue [1,5]. Inhibition of target activity may be achieved using light-sensitive chloride channels or proton pumps (e.g., NpHR3 [6], ArchT [7]), a feat that is more complex with electrical stimulation. Additionally, stimulation of specific downstream nerve targets may be achieved through targeted virus injection, genetic targeting, or opsin selection [8,9]. Taken together, these traits present attractive benefits for the use of this technology in neuroscience or motor rehabilitation applications.

A key step for using this technology is to first biologically label target nerve axons with the desired opsin. Transgenic animals may be used for robust opsin labeling of genetically specified tissues throughout the body, including peripheral nerves for neuromodulation [1,10]. However, transgenic approaches are typically limited to mice, and specificity through these approaches is usually defined by genetic markers, resulting in the labeling of all tissues in the body that meet genetic criteria. Conversely, injection of viral vectors (e.g., adeno-associated virus, AAV) may be used to express optogenetic proteins targeted to tissues based on 1) the relative tropism of the virus (i.e., AAV3 for inner ear cells [11]), 2) genetic specificity tailored by the virus’s expression cassette, and 3) viral biodistribution guided by the route of virus delivery (e.g., broad expression via intravenous injection, focal expression via microinjection in the targeted tissue). These tools provide several knobs through which to tune the specificity or breadth of opsin expression.

In the case of peripheral optogenetic stimulation of motor function, muscle-specific opsin expression and stimulation can be achieved via intramuscular virus injection. For example, intramuscular injection of an AAV6 vector (e.g., AAV6-hSyn-ChR2-eYFP) into the tibialis anterior (TA) muscle of rats can be used to selectively label nerve axons innervating the TA with a light-sensitive opsin (e.g., channel-rhodopsin, ChR2) and a fluorescent tag (e.g., YFP) [2,3]. The human synapsin promoter (hSyn) restricts expression to neural tissue (as opposed to expression in the injected muscle). To label targeted deep peroneal nerve axons innervating just the TA muscle, a primary route would require that virus first crosses from the muscle into the nerve via the neuromuscular junction (NMJ), travel retrogradely up the nerve to motor neurons in the spinal cord, undergo transcription of the viral DNA instructions, and traffic expressed opsin and fluorescent proteins anterogradely back down the nerve axons. Following this injection route, optical stimulation at proximal nerve locations with multiple downstream muscle targets, such as the sciatic nerve, results in stimulation of only the targeted TA muscle [3], a feat of specificity that is challenging with electrical nerve stimulation.

A critical point in this viral transduction pathway is the uptake of the virus from the muscle into the nerve. NMJs are typically arranged in characteristic patterns within a muscle where nerve endings branch out to interface with muscle fibers. Targeting microinjections of virus to follow these NMJ distributions has been shown to increase the uptake of virus and other tracers [12,13]. While injection sites within a muscle may be based on expected NMJ distributions, intersubject variability could potentially limit the success of this approach, especially in larger animals. As an alternative approach, low-level electrical stimulation through intramuscular injection electrodes can be used to localize motor endplates in target muscles. Such an approach has been utilized in human subjects for guiding injections of botulinum toxin, in conditions such as juvenile cerebral palsy, as a fast and minimally invasive way to confirm injection of the targeted muscle and facilitate the paralytic effects of the injection [14,15]. We have previously employed a similar approach for intramuscular AAV injections in non-human primates to achieve optical stimulation of muscle activity [4,16].

Here, our primary purpose is to present a protocol to target AAV injections to motor endplates in hindlimb muscles of rats using electrical stimulation in order to express light-sensitive opsins along their corresponding nerves, thereby allowing light-based stimulation of muscle activity. Following these injections, we describe our common procedures for periodically evaluating a nerve’s optical sensitivity using transdermal laser stimulation to estimate and track the longitudinal time course of functional opsin expression. Finally, we present our standard histology preparations for examining the underlying patterns of opsin expression in nerve and spinal cord samples at the terminal end point of an experiment. These protocols provide a reliable means of achieving robust opsin expression in peripheral motor nerves and optically modulated motor responses. Additionally, these protocols can act as a framework for evaluating the functional opsin expression timelines and histological characteristics of optogenetic vectors with varying serotypes, promoters, and opsins.

## Materials and reagents

**Biological materials**

1. Fischer rat (Charles River, strain 403, 100–200 g, generally 6–8 weeks, male or female)

2. AAV6-hSyn-ChR2(H134R)-EYFP [Virovek, 2E + 13 viral genomes per mL (vg/mL) in 1× PBS buffer containing 0.001% pluronic F-68 and 0.22 μm filter sterilized; storage: -80 °C, maximum one freeze-thaw cycle]

**Reagents**

1. Fast Green FCF (Fisher Scientific, catalog number: BP123-10), storage: 25 °C

2. Sterile water for injection (Fresenius Kabi, catalog number: 918510), storage: 25 °C

3. Loxicom (meloxicam) injection, 5 mg/mL (Norbrook Laboratories, catalog number: NDC 55529-040-10), storage: 25 °C

4. Povidone iodine prep solution (Dynarex Corporation, catalog number: NDC 67777-141-50), storage: 25 °C

5. Beuthanasia-D Special pentobarbital sodium and phenytoin sodium injection solution (Intervet Inc, catalog number: NDC 0061-0473-05), storage: 25 °C

6. OCT compound (Tissue-Tek, catalog number: 4583), storage: 25 °C

7. Molecular Probes ProLong Diamond Antifade Mountant (Fisher Scientific, catalog number: P36961), storage: 4 °C

8. Phosphate-buffered saline (PBS) (Midwest Scientific, catalog number: QS1200), storage: 25 °C

9. 4% paraformaldehyde (PFA) (Electron Microscopy Sciences, catalog number: 157-4), storage: 25 °C

10. Sodium azide (Sigma-Aldrich, catalog number: S2002), storage: 25 °C

11. Sucrose (Sigma-Aldrich, catalog number: S7903), storage: 25 °C

12. Rabbit anti-GFP antibodies (Thermo Fisher Scientific, catalog number: A11122), storage: 4 °C

13. Donkey anti-rabbit antibodies, AF647 conjugated (Jackson ImmunoResearch, catalog number: 711-607-003), storage: -80 °C

14. Normal donkey serum (Sigma-Aldrich, catalog number: D9663), storage: -20 °C

15. Triton X-100 (Thermo Fisher Scientific, catalog number: A16046.AP), storage: 25 °C

**Laboratory supplies**

1. Screw-cap microtubes, 1.5 mL, skirted (Thermo Fisher Scientific, catalog number: 3467-11)

2. Screw-cap microtubes, 0.5 mL, skirted (Thermo Fisher Scientific, catalog number: 3465)

3. Sterile syringe filters, 0.22 μm pore size, mixed cellulose ester membrane (Sigma-Aldrich, catalog number: SLGL0250S)

4. Disposable subdermal needle electrode, XS (Technomed, catalog number: TE/S50718-002; for EMG recording)

5. Disposable hypodermic needle electrode (Technomed, catalog number: TE/L2530-335; for stimulation during virus injection)

6. Covidien monoject hypodermic needles with aluminum hub (Covidien, catalog number: 8881200433; ground connection for EMG recording)

7. ETL Series: single alligator clips (The Electrode Store, catalog number: ETL-36BSAF; ground connection for EMG recording)

8. SGE gas-tight Teflon luer lock syringe, 50 μL (World Precision Instruments, catalog number: SGE050TLL)

9. Microscope cover glass (Midwest Scientific, catalog number: 1415-15)

10. Disposable base mold (Electron Microscopy Sciences, catalog number: 62352-07)

11. Tissue Path Superfrost Plus Gold slides (Fisher Scientific, catalog number: 1518848)

12. Animal feeding needle, 20 G straight (Pet Surgical, catalog number: AFN2025S; for perfusion)

## Equipment

1. Micropositioner and magnetic stand (World Precision Instruments, catalog number: 1350M)

2. MICRO2T SMARTouch pump controller (World Precision Instruments, catalog number: MICRO2T)

3. UMP3 syringe pump (World Precision Instruments, catalog number: UMP3)

4. Nexus optical breadboard (Thorlabs, catalog number: B1824F)

5. LRD-0470 Collimated diode laser system, typical maximum output 200–250 mW at 470 nm, or 600 mW/mm^2^ at 1 mm from 200 μm, 0.22 NA optical fiber–based on a near-field approximation (Laserglow Technologies, catalog number: D4B2003FX)

6. Optical fiber, 200 μm, 0.22 NA FC/PC-FC/PC fiber patch cable (Thorlabs, catalog number: M122L05)

7. Optical power and energy meter console (Thorlabs, catalog number: PM400)

8. Slim photodiode power sensor (Thorlabs, catalog number: S130C)

9. Electrophysiology system with EMG recording and electrical stimulation capabilities (e.g., Tucker-Davis Technologies, RZ5D, SI-4, and S-Box)

10. Custom 3D-printed leg support (design available upon request)

11. Peristaltic pump (Kamoer, catalog number: KK1800)

12. Precision pump tubing, peroxide-cured silicone (Masterflex, catalog number: 96400-16)

13. Polycarbonate Luer-to-Barb adapter 1/8” hose barb (any)

14. Confocal microscope (e.g., Leica Microsystems, model: TCS SP8)

## Procedure

**A. Viral preparation**

**Caution:** This procedure utilizes adeno-associated virus. Follow appropriate BSL-1 safety protocols when working with viruses.

1. Prepare a 1% Fast Green FCF solution.

a. Add 0.1 g of Fast Green to 0.9 mL sterile water for injection in a sterile 1.5 mL microtube to create a 10% Fast Green stock solution, which may be used to make further dilutions. Vortex thoroughly to ensure mixing.

b. Dilute this solution into a 1% Fast Green working solution. Add 100 μL of 10% Fast Green solution and 0.9 mL sterile water to a new 1.5 mL microtube and mix thoroughly.

c. Draw up 1% Fast Green solution into a 1 mL syringe. Attach a sterile 0.22-µm syringe filter to the syringe via luer lock. Depress the syringe plunger until the Fast Green solution passes through the filter into a new sterile microtube. This final solution will be used later during the intramuscular injection surgery to assist with visualization of the virus solution.

d. Add 20 μL of virus solution and 2 μL of filtered 1% Fast Green to a 0.5 mL microtube. Vortex the solution to mix and centrifuge briefly to force the liquid to the bottom of the tube. This solution can be stored for up to 8 h at 4 °C prior to use in virus injection surgery.

*Note: This solution should be prepared on the day of intramuscular injection surgery. Virus should be aliquoted and stored at -80 °C until used, avoiding multiple (more than one) freeze-thaw cycles.*

**B. Intramuscular injection**

1. Clean the surgical table (magnetic optical table) and place a sterile drape on top of an animal warming pad. Set the warming pad to maintain the animal body temperature at 37 °C. Sterilize all surgical tools and place them on a sterile surface near the surgical area.

2. Prepare the syringe and injection needle with the virus solution. Attach a 30 G hypodermic needle electrode to a 50 μL microsyringe. Draw 22 μL of virus solution into the syringe, then depress the plunger until the solution is visible to verify that there is no air in the needle.

3. Connect the syringe pump to the controller. Choose the appropriate syringe (50 μL, 60 mm) and injection (22,000 nL at 5 μL/min) settings. Attach the prepared syringe with the virus solution to the syringe pump.

4. Attach the syringe pump to a magnetic micropositioner and place it out of the way.

5. Place the animal in the induction box and anesthetize using an isoflurane anesthesia system (3% isoflurane in room air, 500 mL/min) until no response to a toe-pinch reflex is observed.

6. Transfer the animal to the nose cone mask for maintenance anesthesia (1.5%–2% isoflurane, 350 mL/min). Place the animal on a disposable pad and remove hair from the lateral surface of the left leg using an electric hair shaver or hair removal cream.

7. Move the animal to a sterile drape, placing the animal on its right side.

8. Prepare the animal for surgery.

a. Apply lubricant eye ointment.

b. Administer subcutaneous (SC) injections of meloxicam (1–2 mg/kg) or other analgesic and lactated Ringer’s solution (10 mL/kg) for fluid maintenance.

c. Place a support underneath the left leg such that the tibia is parallel to the surgical table. Secure the ankle with tape.

d. Scrub the surgical site with povidone iodine solution followed by 70% ethanol. Repeat this process three times.

**Figure 1.**
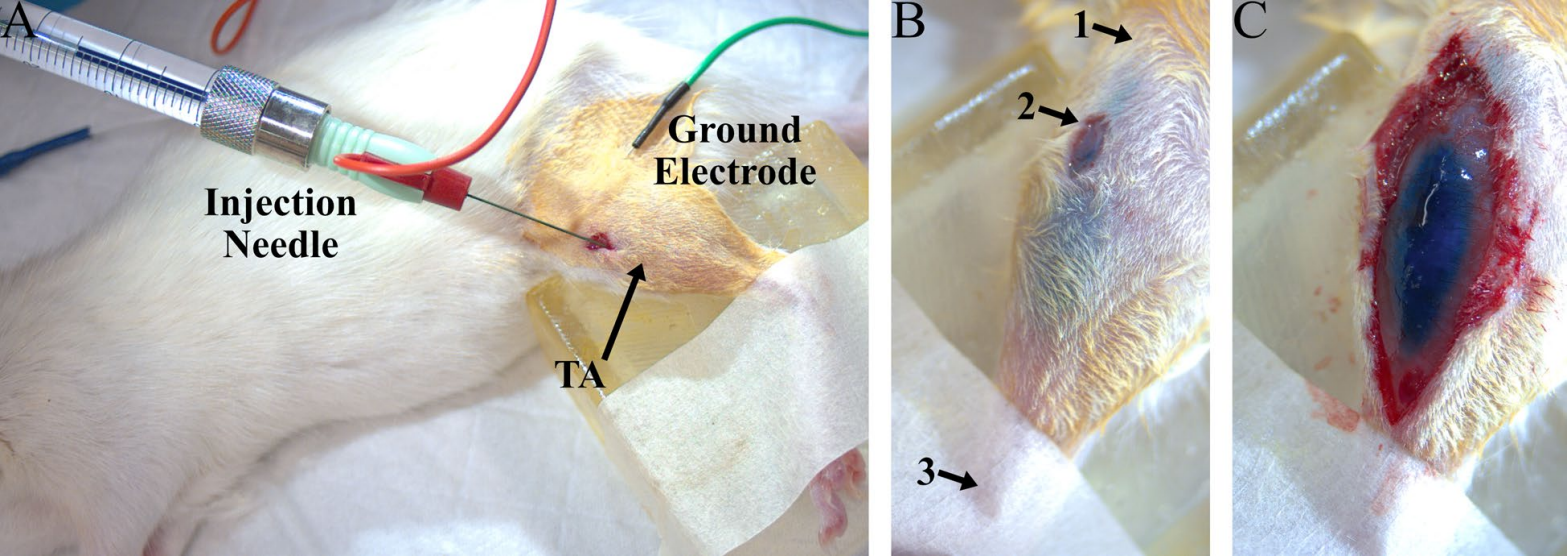
Experiment setup for intramuscular virus injection into the left tibialis anterior (TA). (A) Example photo of a rat virus injection procedure, including the injection needle inserted into the TA (red) and the electrical ground needle placed subcutaneously (green). (B) Photo of a TA muscle after virus injection. The Fast Green dye is clearly visible in the TA through the skin and does not spread beyond the boundaries of the muscle, indicating that the virus was delivered to the desired muscle. Arrows point to approximate landmarks to guide needle placement: 1) proximal end of tibia, 2) location of injection incision, and 3) distal end of tibia. (C) Photo of an exposed TA muscle after virus injection.

9. Make a small (3–5 mm) incision 2–3 mm lateral to the tibia and approximately 5 mm distal to the knee joint to expose the left tibialis anterior (TA) muscle (Figure 1A). Use blunt dissection to separate the skin from the fascia around the incision.

10. Place one needle electrode under the skin on the left leg approximately 1–2 cm away from the TA muscle incision. This wire will serve as the ground of the electrical stimulator.

11. Position the micropositioner with the virus injection syringe for insertion into the muscle in the center of the previously made incision, aiming to enter at the proximal end of the muscle, about one quarter of the distance between the knee and ankle joints. The needle course should run parallel with the TA muscle fibers in the longitudinal plane and enter the muscle at approximately a 30° angle from the muscle surface, pointed toward the distal muscle end (Figure 1A).

12. Connect the electrode side of the syringe needle to the stimulation channel of the electrical stimulator. Connect the needle electrode to the ground channel of the electrical stimulator. Choose electrical stimulation settings (e.g., monopolar, biphasic waveform with 0.2 ms pulse width, 1 Hz frequency).

13. Use the micropositioner to advance the syringe and needle until it enters the muscle. Support the leg as needed to prevent movement while inserting the needle (i.e., hold the muscle and skin taught with your fingers until the needle pierces the muscle).

14. Turn on electrical stimulation and adjust the current until small muscle twitches are visible, typically in the range of 75–300 µA. The magnitude of the twitches should vary with changes in needle position.

15. Adjust needle position until muscle twitches elicited by electrical stimulation are maximized to target the needle position to zones of dense NMJs (distal to the knee joint, about one third to one half of the total distance between the knee and the ankle; Figure 1A, B). Turn off electrical stimulation.

16. Inject 22 μL of virus (5 μL/min) as one continuous injection using an automated microinjection pump. During the injection, the needle can be retracted a small amount every 5 µL to prevent backflow. Wait at least 2 min after the injection has finished to allow any solution remaining in the needle to disperse, then slowly retract the syringe needle from the muscle and remove ground electrode. The viral solution should be confined to the TA muscle to avoid off-target effects (Figure 1B, C). Virus distribution should be highlighted by Fast Green FCF mixed in the virus solution.

17. Suture the incision using 4–0 to 5–0 suture.

18. Remove the animal from anesthesia, return them to their home cage to recover, and monitor until fully awake. Provide postoperative meloxicam (1–2 mg/kg, SC once daily) or other analgesia for three days following the procedure.

**C. Periodic transdermal assessment**

**Caution:** This procedure utilizes Class 3B lasers for optogenetic stimulation. Avoid direct eye exposure to the beam.

*Note: Begin assessment procedures as early as one week after viral injection and repeat periodically until the desired end timepoint of the experiment (i.e., 6–20 weeks).*

1. Power on the electrophysiology equipment and set up connections for EMG recordings. Recording should be set up for differential measurements with a low-impedance headstage, gain of 1, and AC coupling to eliminate any baseline drift in the signal. The signal should be sampled at a frequency to allow for visualization of spikes (e.g., 12.2 kHz).

2. Prepare the laser and verify the output light intensity.

a. Connect one end of an optical fiber (200 μm, 0.22 NA) to the 470 nm laser. Attach the free end of the fiber to a probe holder and place it in the micropositioner.

b. Position the micropositioner such that the laser output is 2–3 mm above the light sensor. Connect the sensor to the power meter and power on. Set the wavelength to 470 nm.

c. Turn on the laser for continuous output at maximum output power. If needed, adjust the optical fiber alignment until the desired light output is obtained.

d. Record the maximum light output. Change the light intensity to the minimum value that will be used during the experiment and record that value.

**Figure 2.**
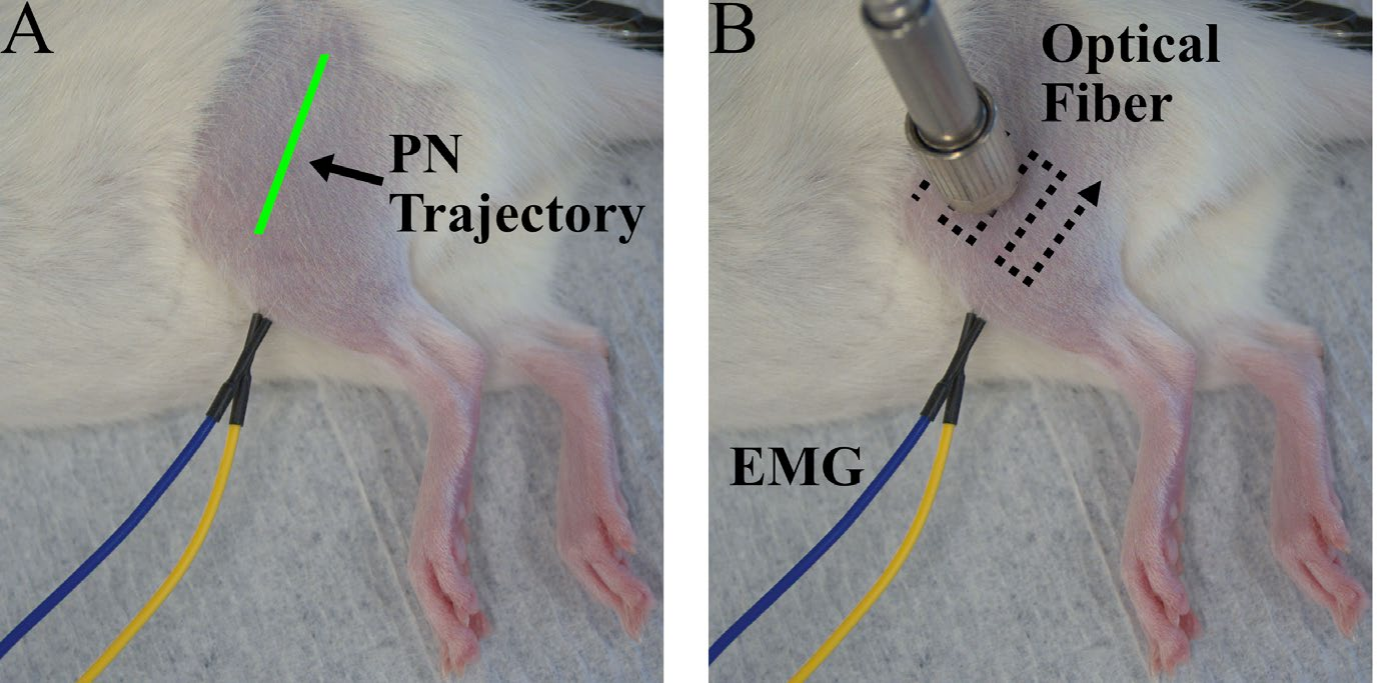
Experiment setup for periodic transdermal assessment sessions. (A) Photo of a rat during a transdermal assessment session prior to optical fiber positioning. The estimated trajectory of the peroneal nerve (PN) is shown in green. (B) Photo of a rat during a transdermal assessment session including EMG electrodes placed in the left tibialis anterior (TA) and optical fiber positioned over the peroneal nerve. A typical optical fiber scanning pattern for optical fiber placement is shown by the dotted lines.

3. Place the animal in the induction box and anesthetize using an isoflurane anesthesia system (3% isoflurane) until no response to a toe-pinch reflex is observed.

4. Transfer the animal to a nose cone mask for maintenance anesthesia (1.5%–2%). Place the animal on its right side on a warming pad and remove the hair from the lateral surface of the left leg.

5. Insert two needle electrodes approximately 2 mm apart along the long axis of the TA muscle for EMG recordings. Electrodes should be inserted at an angle of approximately 45° to the skin surface such that the entirety of the electrode length (7 mm) lies within the muscle. Connect these electrodes to the recording system.

6. Insert a metal hub needle subcutaneously in the animal’s back and connect an alligator clip to the hub of the needle. This will serve as ground for the EMG recordings.

7. Position the micropositioner with the laser attached just above the leg (approximately 0–1 mm) in the approximate position of the peroneal nerve. This nerve is located lateral to the TA muscle about one third of the way between the knee and ankle (Figure 2A).

8. Turn on pulsed optical stimulation of the laser (1 Hz, pulse width 5 ms, maximum light intensity) and observe the EMG signal while continuously scanning the laser position along the leg, moving the laser a small amount with each stimulation pulse. Adjust the laser position until the peak-to-peak magnitude of the recorded EMG spikes is maximized (Figure 2B). In general, if functional opsin expression is present, responses will appear as consistent, time-locked EMG spikes 4–8 ms after each stimulation pulse at 1 Hz. See the Troubleshooting section for information regarding responses that disappear with repeated stimulation. EMG spikes observed out of phase with optical stimulation or those that arise from stimulation far from the target nerve location are likely not due to opsin expression.

9. Run the desired optical stimulation sequences. In general, these can include pulse trains with varying light intensity, stimulation pulse width, and stimulation frequency to assess muscle responses to optical stimulation at the testing time point. Three example stimulation sequences are described below.

a. Light intensity sweep

i. Optical power: 20–240 mW (stimulate at 10–15 values over this range).

*Note: As this protocol uses transdermal stimulation for longitudinal optical stimulation, a more powerful laser (e.g., 100–200 mW) may be required to elicit responses than that required for typical brain or exposed nerve stimulation experiments.*

ii. Stimulation pulse width: 5 ms

iii. Frequency: 1 Hz

iv. Number of pulses: 10 per stimulation train for each light intensity

b. Pulse width sweep

i. Optical power: 240 mW

ii. Stimulation pulse width: 0.5–10 ms (stimulate at 10 values over this range)

iii. Frequency: 0.5 Hz

iv. Number of pulses: 10 per stimulation train

c. Frequency sweep

i. Optical power: 240 mW

ii. Stimulation pulse width: 5 ms

iii. Frequency: 1–75 Hz (stimulation at multiple values over this range)

iv. Stimulation train duration: 2 s at each frequency. Note that a longer break (at least 5 s) should be implemented between each 2-s train.

10. Remove electrodes and ground needle.

11. Remove the animal from anesthesia and monitor until fully awake.

12. Perform data analysis of EMG recordings. Relevant data analysis information is shown below.

a. Response magnitudes can be calculated as the root mean square (RMS) of the recorded EMG signal over a 15 ms window starting at the onset of each optical stimulation pulse. Response magnitudes should be averaged over 5–10 trials.

b. In general, muscle responses will be clearly visible over noise recorded on the EMG. When determining the threshold for a response, EMG spikes should occur in response to at least 75% of optical stimulation pulses, and the absolute peak values should be at least 4 times larger than the noise floor RMS calculated from a time period with no stimulation.

**D. Transcardial perfusion**

**Caution**: This procedure utilizes hazardous chemicals, including PFA and sodium azide. Take appropriate precautions when working with hazardous chemicals.

1. Administer pentobarbital solution (150–200 mg/kg) IP to euthanize the animal. Monitor the animal until no responses to corneal or toe-pinch reflexes are present.

2. Open the chest by cutting the ribs on both sides of the sternum from the diaphragm to the top of the chest, forming a flap. Lift the flap with hemostats to expose the heart.

3. Make a small cut into the left ventricle and insert a blunt 20G needle connected to a perfusion pump into that incision toward the aorta. Use hemostats to secure the needle in place.

4. Make another incision into the right atrium to allow for fluid to leave the system during perfusion.

5. Perfuse the animal with 200 mL of cold 1× PBS at approximately 80 mL/min followed by 200 mL of cold 4% PFA in PBS.

6. Collect the injected muscle and corresponding nerve (TA, peroneal nerve) as well as the spinal cord for immunohistochemistry analysis as described below. The TA muscle is not crucial for expression analysis and is mostly used to keep track of the targeted nerve upon initial harvest. Store spinal cord samples in 4% PFA at 4 °C overnight and nerve samples in 4% PFA for 15 min.

a. Peroneal nerve and TA:

i. Make a large incision on the lateral side of the leg and perform blunt dissection of the skin to expose the TA and biceps femoris (BF) muscles.

ii. Make another incision in the fascia between TA and BF; this should expose the peroneal nerve. Perform blunt dissection as needed to mobilize the nerve.

iii. Follow the peroneal nerve back until it joins with the sciatic nerve, retracting muscles and performing blunt dissection as needed. In general, it may be helpful to detach the BF at the knee joint and reflect that muscle to expose the entire length of the peroneal and sciatic nerves at one time.

iv. Cut the sciatic nerve at the location of the proximal end of the femur and any other connections not including the peroneal nerve (tibial and sural nerves) such that the nerve is only attached to the TA and smaller anterior compartment muscles near the TA.

v. Cut the tendon attachment of the TA at the distal end of the tibia, then remove the TA and accessory muscles by cutting underneath them along the tibia and fibula. Make sure to avoid cutting the nerve attachment to the muscles.

vi. Cut the TA attachment at the knee joint. At this point, the nerves and attached muscles should be completely detached from the rest of the leg.

b. Spinal cord:

i. Make a skin incision from the head to the pelvis on the dorsal side of the animal.

ii. Cut along both sides of the spinal column from L6 extending rostrally to the desired spinal level. To ensure that the entirety of the lumbar cord is collected, extend the sample to at least mid-thoracic vertebral levels.

iii. Cut the spinal column at the rostral and caudal ends.

7. Post-fix the spinal column in 4% PFA overnight. Perform a laminectomy to expose and remove the spinal cord and dorsal root ganglia (DRG). Alternatively, perform the laminectomy without removing the entire spinal column. Transfer samples to 1× PBS with 0.02% sodium azide and store at 4 °C. Samples should be frozen and sliced as described below (Section E) within 4–6 weeks for optimal imaging.

**E. Immunohistochemistry (IHC)**

1. Dissect a section of the target peroneal nerve or spinal cord for IHC and transfer to a 20% sucrose solution in PBS overnight at 4 °C to prepare tissue for freezing.

2. Freeze tissue in OCT using liquid nitrogen or another fast-freezing method.

3. Slice tissue into cross sections (14–30 μm) using a cryostat.

4. Wash samples with PBS for at least 5 min. Repeat three times. If needed, the fluorophore signal may be amplified using IHC as follows (optional):

a. Block samples in incubation buffer (1% normal donkey serum, 1% Triton X-100 in PBS) for 30 min at room temperature.

b. Wash samples with PBS for 5 min. Repeat three times.

c. Incubate samples with primary antibodies (rabbit anti-GFP, 1:200) overnight at 4 °C. The antibodies should be diluted with the same incubation buffer used in step E4a. Note that primary and secondary antibodies can be modified for use with other fluorophores.

d. Wash samples with PBS for 5 min. Repeat three times.

e. Incubate samples with secondary antibodies (donkey anti-rabbit conjugated to AF647, 1:100) for 2 h at room temperature. These antibodies should be diluted using the incubation buffer used in step E4a.

f. Wash samples with PBS for 5 min. Repeat three times.

5. Mount coverslips to slides with mounting media.

6. Image slides using a confocal microscope to visualize opsin expression. Choose imaging settings appropriate to the fluorophore co-expressed with the opsin or secondary antibody used during IHC.

## Validation of protocol

Viral-mediated optogenetic stimulation of peripheral nerves is a promising neuromodulation technique for motor rehabilitation after spinal cord injury. However, achieving sufficient opsin expression in targeted nerves to modulate neural activity and drive functional movements remains a challenge. Additionally, opsin expression changes over time after injection, necessitating a method for assessing functional opsin expression at multiple time points in the same animal. In this protocol, we describe methods for achieving robust opsin expression in a specific target nerve via stimulation-guided intramuscular viral injection, repeatable and non-invasive functional assessment of opsin expression, and immunofluorescence visualization of opsin expression.

The use of electrical stimulation during the intramuscular virus injection surgery serves as a method to ensure that the injection needle is placed near neuromuscular junctions in the target muscle for a particular subject. Performing injection close to these endplates can increase viral uptake, leading to increased transgene expression [13]. The movements produced by muscle activation during stimulation can also verify that the needle is placed in the correct target muscle prior to injection. The Fast Green dye allows for visualization of the virus, even through the skin, and can further confirm the success of a virus injection (Figure 1B, C).

**Figure 3.**
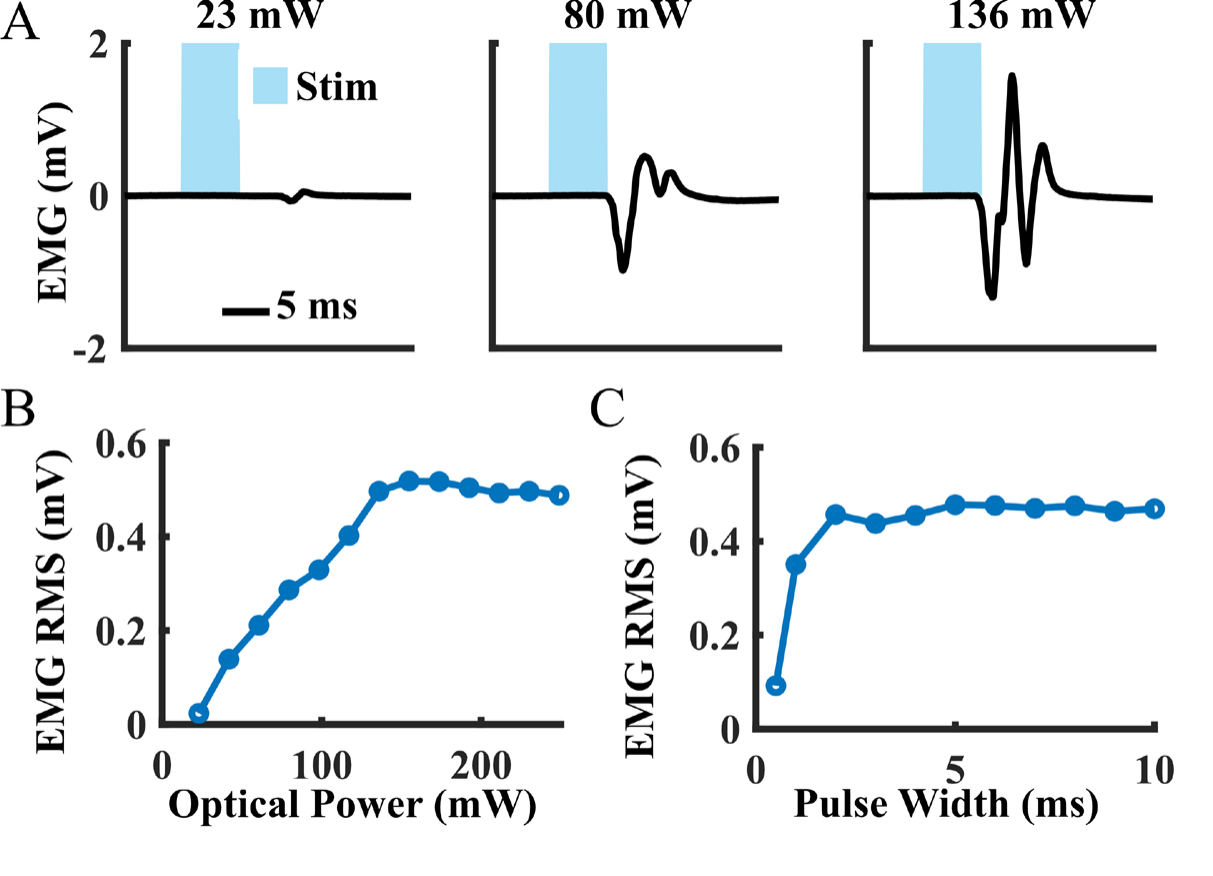
Example muscle activity data from a periodic transdermal assessment session at six weeks after intramuscular tibialis anterior (TA) virus injection. (A) Mean electromyography (EMG) waveforms recorded from the TA during single pulses of transdermal optical peroneal nerve stimulation. (B) Average EMG response magnitudes to 1 Hz optical stimulation over a range of light intensities (pulse width: 5 ms). Magnitudes were calculated as the root mean square (RMS) of the recorded EMG signal over a 15-ms window beginning at the onset of each optical pulse. (C) Average EMG response magnitudes to 0.5 Hz optical stimulation over a range of stimulation pulse widths (optical power: 250 mW).

**Figure 4.**
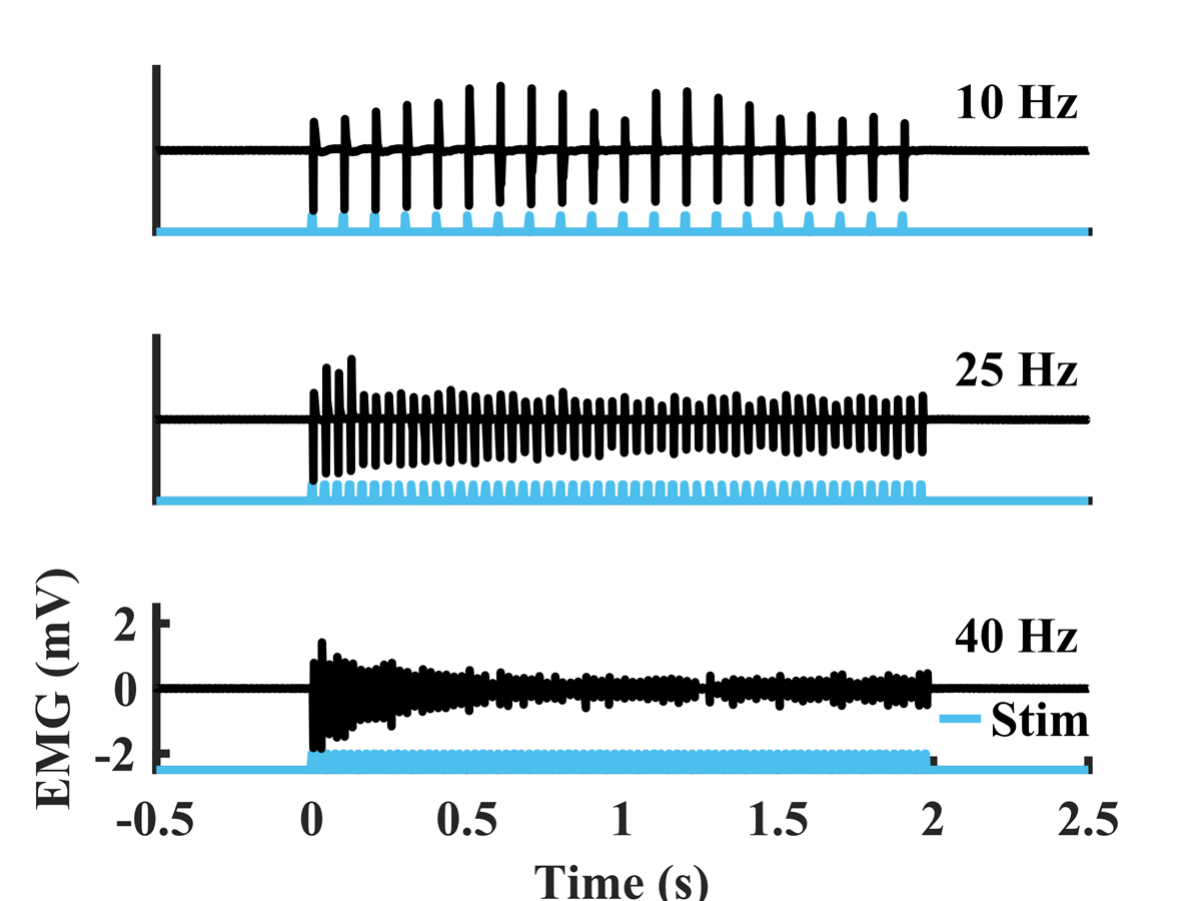
Example electromyography (EMG) recorded from the tibialis anterior (TA) during sustained transdermal optical peroneal nerve stimulation trains over a range of frequencies (duration: 2 s, pulse width: 5 ms, optical power: 250 mW) from a rat at six weeks after virus injection

After intramuscular virus injection, functional optical sensitivity of the target nerve can be evaluated with periodic transdermal optical stimulation sessions. The periodic transdermal optical stimulation assessment sessions described in this protocol are one way to non-invasively examine electrophysiological and functional responses to optical stimulation at multiple timepoints in a single animal. If functional opsin expression is present, transdermal optical stimulation will result in time-locked EMG spikes 4–8 ms after each optical stimulus pulse (Figure 3A). In our experience, the onset of functional optical sensitivity generally occurs 2–4 weeks after virus injection and peaks between 6 and 10 weeks, though this may vary depending on the virus serotype and opsin used, as well as the target muscle, age and size of animal, and species. For example, we have observed comparable expression timelines and strength using AAV-retro rather than AAV6 in adult rats. However, injection of larger animals such as non-human primates may result in an increased delay between virus injection and observable functional opsin expression [4]. Both light intensity and stimulus pulse width modulate the magnitude of stimulation-driven muscle responses (Figure 3B, C). The light intensity threshold to evoke a muscle response, the effective linear working range of stimulation, and the size of movements driven by optical stimulation will vary between sessions as the amount of expressed opsins accessible at the stimulation site changes over time. The magnitude of muscle responses to higher frequency optical stimulation will decay over the course of a longer stimulus train (Figure 4). This behavior is most likely due to frequency limitations of opsin kinetics, as ChR2 has relatively slow open/close rates [17]. However, the extent to which this decay occurs is also variable between optical stimulation sessions due to evolving opsin expression levels. Muscle activation via transdermal optical stimulation of the peroneal nerve has been previously demonstrated in neonatal rats [2]. We have expanded upon this technique by performing a set of transdermal stimulation sequences with a variety of parameters such as light intensity, pulse width, and stimulation frequency. The goal of these sequences is to provide additional characterization of muscle responses to stimulation rather than a purely binary metric provided by the presence or absence of muscle twitches in response to optical stimulation. Using this method, muscle activity recorded also operates as a proxy for IHC measurements of opsin expression, as only one IHC measurement can be collected per animal.

**Figure 5.**
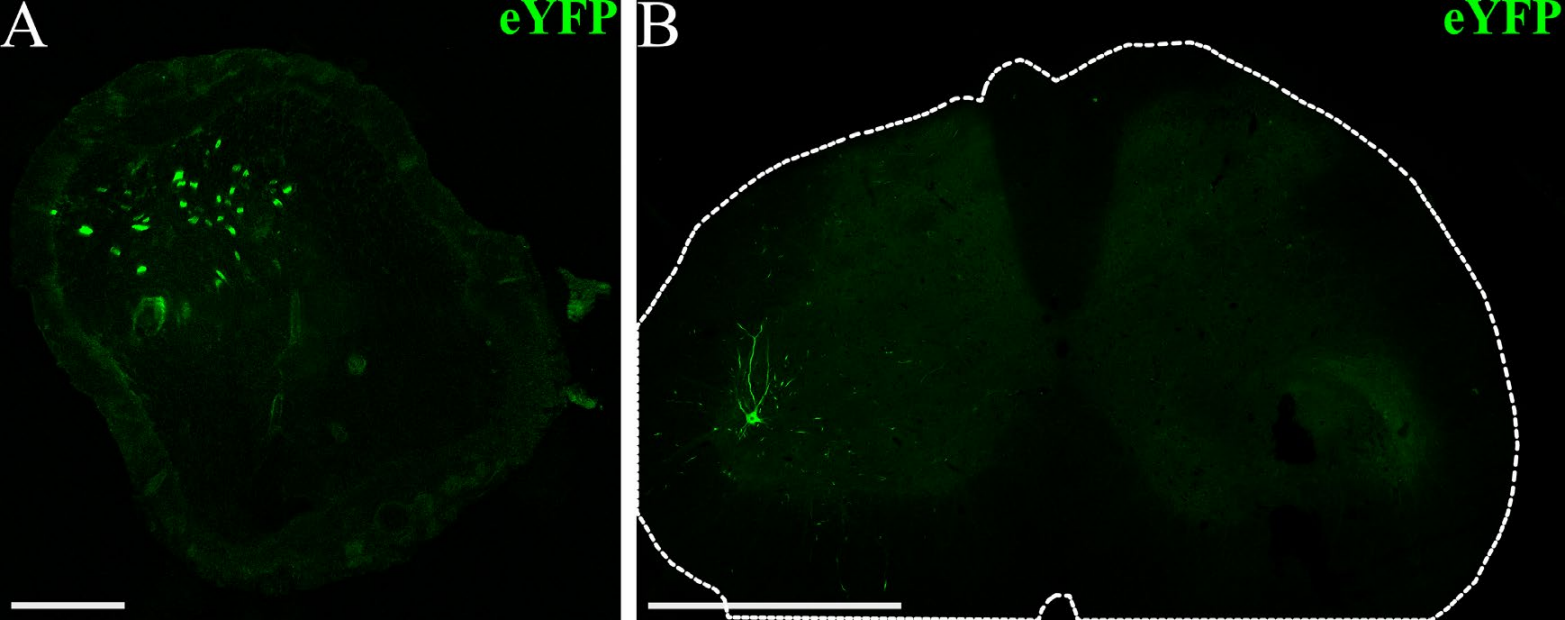
Example visualization of opsin expression using immunohistochemistry (IHC). (A) Confocal image of a peroneal nerve cross-section (scalebar: 100 μm) from a rat that received an intramuscular virus injection in the left tibialis anterior (TA) and lateral gastrocnemius (GN) muscles (AAV6-hSyn-ChR2-eYFP). The fluorescence signal corresponds to ChR2-eYFP expression at the conclusion of the experiment, seven weeks after virus injection. (B) Confocal image of the lumbar spinal cord (scale bar: 1 mm) from a virus-injected rat showing ChR2-eYFP expression in motor neurons and projections in the ventral horn. The outer edge of the spinal cord is outlined in white.

Opsin expression can be visualized using immunohistochemistry at the conclusion of the experiment if opsins are co-expressed with a reporter fluorophore. In a cross-section of the peroneal nerve, neurons expressing ChR2 will typically appear as bright rings, with fluorescence concentrated in axonal membranes of labeled neurons (Figure 5A). Opsin expression will also likely be present primarily in the ventral horn of the spinal cord, corresponding to motor neurons innervating the injected muscle (Figure 5B). Some additional expression may be apparent in dorsal root ganglion (DRG) neurons and projections, but this is usually sparser than motor labeling with AAV6. Using more restrictive viral promoters may target opsin expression more specifically to motor or sensory neurons, though this may also reduce the strength of opsin expression, as ubiquitous promoters generally drive the most robust expression. Visualization of opsin expression using IHC serves as another method to confirm axonal opsin expression in the target nerve. In summary, the protocol presented here can serve as a reliable method for achieving robust peripheral nerve opsin expression with known viral vectors. It can also serve as an approach for assessing functional opsin expression over time for novel vectors with unknown expression timelines, as the transdermal optical stimulation sessions and IHC provide multiple metrics for assessing opsin expression.

## General notes and troubleshooting

**General notes**

1. The viral doses and experiment timelines for this protocol were optimized for Fischer rats (100–200 g) and TA injections. Other rat strains, sizes, or ages may impact the timeline of opsin expression or require adjustments to the volume of virus injected to achieve sufficient expression.

2. Variation in EMG electrode placement for each transdermal assessment session may result in high variability in the magnitude of raw EMG signals. Other metrics calculated from recorded EMG may be better suited for quantifying performance over time (e.g., light intensity threshold, effective linear working range) as these measurements are based on EMG as a function of the light stimulus, rather than solely the magnitude of EMG responses.

3. This approach can be expanded to include other muscles. However, the viral dose needed to achieve sufficient opsin expression and the timeline of expression may vary between muscles.

4. Opsin expression may be present in both afferent and efferent neurons, as the synapsin promoter does not appreciably distinguish between these neuron types. We have observed some labeled neurons in lumbar DRGs after intramuscular injection with AAV6, in addition to labeled motor neurons in the spinal cord. This injection and transdermal assessment paradigm can also be used with different AAV serotypes and promoters to more selectively target the population of neurons that will express the opsin after injection toward motor or sensory neurons.

**Troubleshooting**

Problem 1: Electrical stimulation during the injection procedure is not producing any visible twitches in the target muscle.

Possible cause: The injection needle is not in close proximity to NMJs.

Solution: Relocate injection needle. If possible, move closer to the estimated position of NMJs.

Problem 2: During a transdermal stimulation session, muscle responses are initially present but disappear with repeated stimulation.

Possible cause: Repeated optical stimulation is pushing expressed opsins into an extended closed state such that stimulation is no longer effective at generating muscle responses. We have most commonly observed this phenomenon when opsin expression in the target nerve is weak.

Solution: Decrease optical stimulation frequency to 0.3–0.5 Hz to allow more time for photocycle recovery in expressed opsins.

Problem 3: Functional responses to optical stimulation were present during transdermal stimulation, but no labeled neurons are seen on IHC.

Possible cause: Weak opsin expression in the target nerve. The fluorescence signal of the co-expressed reporter protein is not sufficient to visualize labeled neurons.

Solution: Perform IHC amplification of the co-expressed fluorophore to visualize labeled neurons.
